# Cross-sectional study of diet, physical activity, television viewing and sleep duration in 233 110 adults from the UK Biobank; the behavioural phenotype of cardiovascular disease and type 2 diabetes

**DOI:** 10.1136/bmjopen-2015-010038

**Published:** 2016-03-15

**Authors:** Sophie Cassidy, Josephine Y Chau, Michael Catt, Adrian Bauman, Michael I Trenell

**Affiliations:** 1 Faculty of Medical Sciences, Institute of Cellular Medicine, Newcastle University, Newcastle upon Tyne, UK; 2 Prevention Research Collaboration, Sydney School of Public Health, Charles Perkins Centre D17, University of Sydney, New South Wales, Australia; 3 Faculty of Medical Sciences, Institute of Neuroscience, Newcastle University, Newcastle upon Tyne, UK

**Keywords:** DIABETES & ENDOCRINOLOGY, EPIDEMIOLOGY, PUBLIC HEALTH, SLEEP MEDICINE

## Abstract

**Objectives:**

Simultaneously define diet, physical activity, television (TV) viewing, and sleep duration across cardiometabolic disease groups, and investigate clustering of non-diet lifestyle behaviours.

**Design:**

Cross-sectional observational study.

**Setting:**

22 UK Biobank assessment centres across the UK.

**Participants:**

502 664 adults aged 37–63 years old, 54% women. 4 groups were defined based on disease status; ‘No disease’ (n=103 993), ‘cardiovascular disease’ (CVD n=113 469), ‘Type 2 diabetes without CVD’ (n=4074) and ‘Type 2 diabetes + CVD’ (n=11 574).

**Main outcomes:**

Diet, physical activity, TV viewing and sleep duration.

**Results:**

People with ‘CVD’ report low levels of physical activity (<918 MET min/week, OR (95% CI) 1.23 (1.20 to 1.25)), high levels of TV viewing (>3 h/day; 1.42 (1.39 to 1.45)), and poor sleep duration (<7, >8 h/night; 1.37 (1.34 to 1.39)) relative to people without disease. People with ‘Type 2 diabetes + CVD’ were more likely to report low physical activity (1.71 (1.64 to 1.78)), high levels of TV viewing (1.92 (1.85 to 1.99)) and poor sleep duration (1.52 (1.46 to1.58)) relative to people without disease. Non-diet behaviours were clustered, with people with ‘CVD’ or ‘Type 2 diabetes + CVD’ more likely to report simultaneous low physical activity, high TV viewing and poor sleep duration than those without disease (2.15 (2.03 to 2.28) and 3.29 (3.02 to 3.58), respectively). By contrast, 3 in 4 adults with ‘Type 2 diabetes’, and 2 in 4 adults with ‘CVD’ have changed their diet in the past 5 years, compared with only 1 in 4 in the ‘No disease’ group. Models were adjusted for gender, age, body mass index, Townsend Deprivation Index, ethnicity, alcohol intake, smoking and meeting fruit/vegetable guidelines.

**Conclusions:**

Low physical activity, high TV and poor sleep duration are prominent unaddressed high-risk characteristics of both CVD and type 2 diabetes, and are likely to be clustered together.

**Strengths and limitations of this study**

- Diet, physical activity, television (TV) viewing and sleep duration were simultaneously investigated in a very large and well-described UK population cohort.
- Cardiometabolic disease groups were compared with disease-free individuals, and show that worsening cardiometabolic health is associated with a progressive unhealthy behavioural phenotype, consisting of low physical activity, high TV viewing and poor sleep duration.
- The cross-sectional nature means we cannot infer causality between disease and lifestyle behaviours.
- Lifestyle behaviours were self-reported, which may limit the accuracy of measurements.

## Introduction

Cardiovascular disease (CVD) and type 2 diabetes represent significant personal, economic and societal burdens. CVD accounts for a quarter of all UK deaths,^1^ and people with type 2 diabetes carry twice the risk of developing CVD.^2^ With over 700 new cases of diabetes diagnosed daily,^3^ total healthcare expenditure on diabetes is forecast to rise from 10% to 17% by 2035.^4^ The inter-relationship between cardiovascular and metabolic disease is termed cardiometabolic health, and reflects their common environmental and genetic antecedents. Those with both CVD and type 2 diabetes have a particularly poor prognosis, and require aggressive risk factor intervention.^5^

Behavioural factors, spanning diet, physical activity, sedentary behaviour and sleep are major risk factors for the development of cardiometabolic disease. The reduction in energy expenditure through (1) lack of physical activity and (2) increase in sedentary behaviours are risk factors for cardiometabolic disease.^6^ ^7^ Indeed, technological advancements of the 21st century have paved the way for sedentary behaviours, such as watching TV, driving and sitting at a computer becoming the ‘norm’ in modern society, so that physical inactivity is now the fourth leading cause of disease and disability in the UK.^8^ An important lifestyle behaviour, but often forgotten, is sleep, and this is strongly linked to cardiometabolic disease.^9^ ^10^ Sleep is vital for resetting homoeostasis and regulating metabolism, yet changes in working patterns and increased demands on time means sleep debt is a growing issue.

Since WHOs global strategy on diet, physical activity and health,^11^ there have been calls for countries to develop national policy approaches to these lifestyle behaviours.^12^ Indeed, in 2011 the UK government published physical activity recommendations,^13^ and Eat Well was produced as a policy tool that defines government recommendations on healthy diets.^14^ Specific National Institute for Health and Care Excellence (NICE) recommendations for CVD and type 2 diabetes recognise the importance of improving physical activity and diet, but guidance on sitting time or sleep behaviours has not been addressed.^15^ ^16^ Nonetheless, knowledge of baseline behaviours in the population are lacking.

The UK Biobank is a large population-based cohort, and allows measurement of important lifestyle behaviours at the same time, at scale and in a well-described cohort. Our primary aim was to observe the differences in lifestyle behaviours simultaneously across cardiometabolic disease. Our secondary objective was to explore clustering of unhealthy non-diet behaviours across disease groups.

## Methods

### Population and study design

A cross-sectional analysis was conducted on baseline data from the UK Biobank. The UK Biobank is a large, population-based cohort study examining the inter-relationships between environment, lifestyle and genes.^17^ Around 9.2 million invitations were mailed to recruit 502 664 adults (response rate 5.5%) aged between 37 and 73 years.^17^ Recruitment occurred between 2007 and 2010, via 22 assessment centres across the UK. During an assessment centre visit, there were six stages; consent, touchscreen questionnaire, verbal interview, eye measures, physical measures and blood/urine sample collection. The touchscreen questionnaire covered sociodemographics, occupation, lifestyle, early life exposures, cognitive function, family history of illness and medical history. Details of procedures have been previously published.^17^ Participant written informed consent was obtained prior to data collection. All data extracted were deidentified for analysis.

### Disease categories

**Figure 1:**
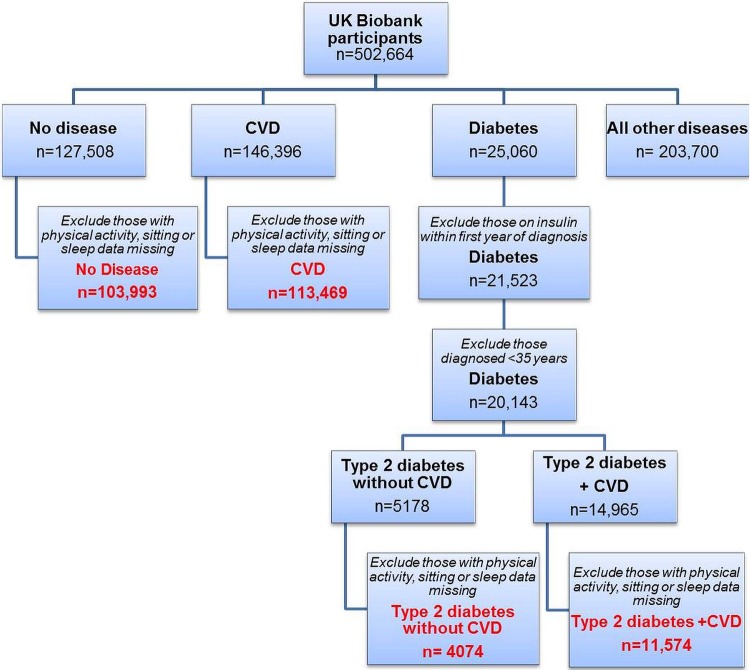
Flow chart demonstrating how disease groups were defined. Final four groups shown in red.

Self-report disease status was obtained from participants during the touchscreen questionnaire, which was then entered and verified by a UK Biobank nurse after further questioning during the verbal interview. We have identified four disease groups spanning cardiometabolic health. (1) *Healthy reference group*: Participants with no disease listed were classified as the ‘No disease’ group. (2) *CVD*: Based on the International Classification of Diseases 10^18^ and a clinician's opinion (AB), diseases to include in the ‘CVD’ group were selected and any patients with type 2 diabetes or diabetes-related comorbidities (including diabetic neuropathy, diabetic nephropathy and diabetic eye disease) were excluded (a list of diseases included in the CVD group can be found in the online supplementary material 1). *Type 2 diabetes*: Participants who were entered as having ‘diabetes’ or ‘type 2 diabetes’ were selected. Those taking insulin within their first year, and were <35 years old at diagnosis were excluded to reduce the likelihood of type 1 and monogenic forms of diabetes. Those without and with CVD were separated into (3) ‘Type 2 diabetes without CVD’ and (4) ‘Type 2 diabetes + CVD’, respectively (figure 1). Excluded from analysis was the ‘Other diseases’ group (n=203 700) with a wide range of diseases covering respiratory, gastrointestinal, renal, neurology, musculoskeletal, haematology, gynaecology, immunological and infectious. A general summary of this group can be found in online supplementary material 2.

10.1136/bmjopen-2015-010038.supp1 Supplementary data

### Baseline measurements

Sociodemographic, smoking, alcohol and dietary intake, physical activity, TV viewing and sleep duration data were collected from the touchscreen questionnaire. Physical activity was assessed using adapted questions from the validated short International Physical Activity Questionnaire (IPAQ)^19^ which covers the frequency, intensity and duration of walking, moderate and vigorous activity. Time spent in vigorous, moderate and walking activity was weighted by the energy expended for these categories of activity, to produce MET min/week of physical activity, which is referred to as ‘total physical activity’. Data processing rules published by IPAQ were followed.^20^ MET.min/week was used for further analysis across disease groups in this manuscript, but we have also included analysis of the separate frequency (days) and duration (min) in online supplementary material 3.

To measure TV viewing, participants were asked ‘In a typical day, how many hours do you spend watching television?’ based on previous literature.^21^ This was asked twice to those who responded >8 h, therefore, high values were deemed robust. To measure sleep duration, participants were asked ‘About how many hours sleep do you get in every 24 h? (please include naps)’. This was asked twice to those who responded >12 h.

Diet intake was reported using the Food Frequency Questionnaire,^22^ in which a number of questions were used based around commonly eaten food groups. Information on fresh/dried fruit, salad and cooked/raw vegetables were combined to create a binary variable to identify individuals who did and did not meet the UK's current guidelines on fruit and vegetable consumption (5/day).^14^ Participants were asked ‘Have you made any major changes to your diet in the last 5 years?’ and were also required to select any of the following foods they ‘Never eat’; eggs, dairy, wheat or sugar. This data was used to observe dietary change and sugar consumption across the four disease groups.

Townsend Deprivation Index was used as a measure of socioeconomic status, by combining census data and postal codes of participants. The index combines information on housing, employment, car availability and social class, with higher values indicating greater deprivation.

Body mass index (BMI) was calculated from: weight(kg)/height(m)^2^. Weight was measured using the Tanita BC-418MA body composition analyser, to the nearest 0.1 kg and height was measured using a Seca 202 height measure. Trained staff took these measures, and participants were required to remove shoes and heavy outer clothing.

### Statistical analysis

All data analyses were performed using SPSS, V.21.0 (IBM, Armonk, New York, USA). Individuals with missing data on total physical activity, TV viewing or sleep duration, were excluded (figure 1). Online supplementary material 4 shows the sociodemographics of missing cases, which were similar to the main cohort, but had a lower proportion of men across all groups. Total physical activity, vigorous, moderate and walking minutes alongside TV viewing were categorised into four groups based on the quartile demarcators for the ‘no disease’ group. Total physical activity groups were labelled as ‘low physical activity’ (lowest quartile: ≤918 MET.min/week) and ‘high physical activity’ (highest quartile: >3706–19 278 MET.min/week) and TV viewing was labelled as ‘low TV viewing’ (lowest quartile: ≤1 h/day) and ‘high TV viewing’ (highest quartile: >3 h/day). As sleep duration shows a ‘U’-shaped relationship with diabetes risk (rather than a linear relationship like physical activity and TV viewing), the data were split using predefined thresholds from the literature; <7 h, 7–8 h and > 8h cut points were used based on a recent meta-analysis.^10^ Sleep duration was labelled as ‘poor sleep duration’ (<7 or >8 h/night) and ‘good sleep duration’ (7–8 h/night). Owing to the large sample size, Pearson's χ^2^ deemed any small difference in group proportions as significant, therefore, these results are not reported.

Non-diet lifestyle behaviours (including physical activity, TV viewing and sleep duration) were further analysed across cardiometabolic disease groups. The proportion of adults reporting all three unhealthy non-diet lifestyle behavoiurs (low physical activity, high TV viewing and poor sleep duration) was calculated for each group and labelled as having an ‘unhealthy phenotype’. By contrast, those who reported high physical activity, low TV viewing and good sleep duration were labelled as having a ‘healthy phenotype’. Binary logistic regression was used to determine the odds of reporting low physical activity, high TV viewing and poor sleep separately, alongside the odds of reporting an ‘unhealthy phenotype’ across disease groups. Additionally, to further investigate the importance of physical activity intensity in cardiometabolic disease, binary logistic regression was used to compare the odds of reporting low walking, moderate and vigorous activity across disease groups. Adjusted ORs, with 95% CIs were reported. All logistic regression models were adjusted for: age (reference=‘40–49’); gender (reference=‘Female’); BMI (reference=‘<18.5–24.9’); Townsend Deprivation Index (reference=‘least deprived); ethnicity (reference=‘White/British); alcohol (reference=‘Never’); smoking (reference=‘Never’); meets fruit/vegetable guidelines (reference=‘YES’). Of the 233 110 cohort, data was missing for; BMI (0.006%), Townsend Deprivation Index (0.002%), ethnicity (0.003%), smoking status (0.003%), alcohol status (0.001%), and fruit and vegetable guidelines (0.015%), therefore, these cases were excluded from the logistic regression models. All statistical tests were two-sided, and significance was set at p<0.05.

## Results

Of the 502 664 UK Biobank participants, after excluding those with missing data or who were likely to have type 1 diabetes, there were 103 993 (21%) with no disease, 113 469 (23%) with CVD, 4074 (1%) with type 2 diabetes without CVD, and 11 574 (2%) with type 2 diabetes + CVD (figure 1). As expected across worsening cardiometabolic disease groups, the proportion of men and those aged >60 years old increased, as did those classified as obese (table 1). There was a marked increase in obesity, with numbers almost quadrupling in the ‘Type 2 diabetes + CVD’ group, compared with disease-free individuals (60.0% vs 15.0%). The ‘No disease’ group had a higher proportion of white/British, and least deprived individuals compared with cardiometabolic diseases. According to the Townsend Deprivation Index, socioeconomic status decreased across cardiometabolic disease groups (table 1).

**Table 1:** Sociodemographics characteristic of disease groups (n=233 110)

	Percentage within each disease group			
	No disease (n=103 993)	CVD (n=113 469)	Type 2 diabetes without CVD (n=4074)	Type 2 diabetes + CVD (n=11 574)
*Sociodemographics*				
% Male	47.0	53.3	63.6	68.0
Age (n), years	103 993	113 469	4074	11 574
37–49	35.6	12.5	13.5	6.0
50–59	35.9	30.2	32.6	27.6
60–73	28.6	57.4	53.9	66.4
BMI (n), kg/m_2_	103 443	112 852	4048	11 478
<18.5–24.9 (under and acceptable weight)	42.9	22.0	14.9	7.2
25–29.9 (overweight)	42.1	44.4	40.5	32.8
≥30 (obese)	15.0	33.6	44.6	60.0
Townsend deprivation quintile (%)	103 861	113 323	4070	11 557
1 (least deprived)	21.9	19.7	17.7	14.7
2	20.8	19.9	17.3	17.4
3	20.7	19.9	18.9	18.5
4	19.6	20.0	20.8	21.0
5 (most deprived)	17.1	20.5	25.1	28.5
Ethnicity (n)	103 687	113 130	4060	11 528
White/British	94.6	95.0	85.6	89.9
Mixed	0.6	0.5	0.6	0.6
Asian	1.8	1.7	8.1	5.1
Black African	1.5	1.8	3.3	2.9
Chinese	0.5	0.2	0.5	0.2
Other	0.9	0.7	2.0	1.3

BMI, body mass index; CVD, cardiovascular disease.

Compared with the ‘No disease’ group, the ‘Type 2 diabetes + CVD’ group reported higher levels of previous smoking (n=5571 (48.3%) versus n=30 960 (29.8%)) and alcohol (n=853 (7.4%) versus n=2020 (1.9%)), but lower current alcohol consumption (n=9891 (85.5%) vs n=98 354 (94.6%)). Dietary data indicate that three-quarters of those with type 2 diabetes have altered their diet within the past 5 years (‘Type 2 diabetes without CVD’: 75.5% and ‘Type 2 diabetes + CVD’: 75.3%), and also half of them never eat sugar (‘Type 2 diabetes without CVD’: 49.8% and ‘Type 2 diabetes + CVD’: 51.2%), which is proportionally more than the ‘CVD’ and ‘No disease’ groups. Around a third of the ‘No disease’ group met the UK's fruit and vegetable guidelines (29.8%) with an increasing trend across cardiometabolic disease (table 2). All other dietary behaviours are reported in online supplementary material 5.

**Table 2:** Lifestyle characteristics of disease groups (n=233 110)

	Percentage within each disease group			
	No disease (n=103 993)	CVD (n=113 469)	Type 2 diabetes without CVD (n=4074)	Type 2 diabetes + CVD (n=11 574)
*Diet*				
Dietary change in past 5 years	103 902	113 300	4070	11 555
YES	28.9	45.9	75.5	75.3
Meets fruit/veg guidelines	102 798	111 554	3995	11 347
YES	29.8	32	35.7	36.6
‘Never eat’	103 848	113 190	4039	11 527
Never eat sugar or foods/drinks containing sugar	14.8	21.0	49.8	51.2
*Physical activity*				
Total physical activity* (MET.min/wk)	103 993	113 469	4074	11 574
≤918 (Low physical activity)	25.0	30.5	35.4	40.1
>918–1902	25.0	24.2	22.5	22.2
>1902–3706	25.0	22.2	20.7	19.7
>3706–19 278 (high physical activity)	25.0	23.2	21.3	18.0
Walking* (min/day)	103 993	113 469	4074	11 574
0–20 (low walking)	31.5	33.9	36.6	40.4
21–30	20.8	20.4	21.0	19.6
31–60	26.7	25.8	23.0	23.6
61–180	21.1	19.9	19.4	16.3
Moderate activity* (min/day)	103 993	113 469	4074	11 574
0–15 (low moderate)	27.8	31.4	36.1	39.6
16–30	28.0	24.7	25.3	23.2
31–60	25.2	23.2	19.9	20.1
61–180	19.0	20.6	18.7	17.1
Vigorous activity* (min/day)	103 993	113 469	4074	11 574
0 (low vigorous)	34.2	46.1	49.7	56.5
1–20	20.3	19.6	19.7	18.0
21–45	21.7	16.7	14.5	13.1
46–180	23.7	17.6	16.1	12.3
Meets UK government physical activity guidelines†	103 993	113 469	4074	11 574
NO	16.1	20.0	24.7	27.5
*TV viewing*				
TV viewing* (h/day)	103 993	113 469	4074	11 574
≤1 (Low TV viewing)	26.6	16.2	15.4	10.5
>1–2	30.5	24.5	22.3	19.3
>2–3	22.6	24.5	24.1	22.8
>3 (High TV viewing)	20.3	34.8	38.1	47.3
*Sleep*				
Sleep duration‡ (h/night)	103 993	113 469	4074	11 574
<7 (Poor sleep duration)	21.3	26.4	27.0	27.5
7–8 (Good sleep duration)	73.4	64.6	62.1	58.6
>8 (Poor sleep duration)	5.3	9.1	10.8	13.9
*Behavioural phenotype*				
UNHEALTHY (low physical activity, high TV viewing and poor sleep duration)	1.8	5.2	5.8	10.0
HEALTHY (high physical activity, low TV viewing and good sleep duration)	4.5	2.4	1.9	1.2

*For physical activity and TV viewing categories, quartiles were calculated from the ‘No disease’ group, so that their demarcators could be applied to disease group.

†UK Government recommendations of 150 min of moderate or 75 min of vigorous activity per week.

‡Physiological thresholds used rather than quartiles because the shape of the risk relationship is a U shape (not linear like physical activity and TV viewing).

BMI, body mass index; CVD, cardiovascular disease.

**Figure 2:**
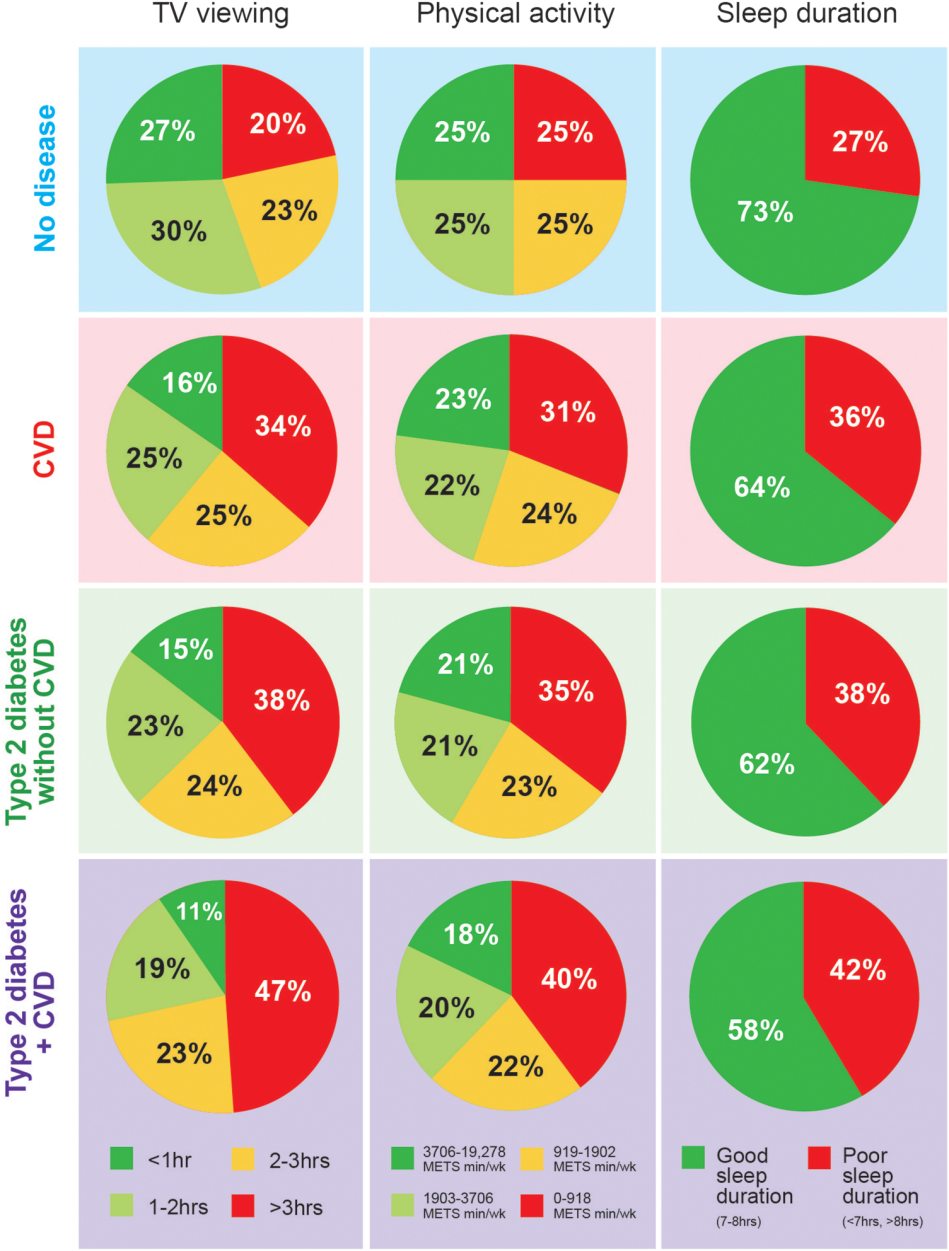
Distribution of physical activity, TV viewing and sleep duration in people with No disease, CVD, Type 2 diabetes without CVD, or Type 2 diabetes + CVD. Red indicates unhealthy and green indicates healthy lifestyle behaviours. CVD, cardiovascular disease.

Total physical activity levels declined across cardiometabolic disease groups (table 2 and figure 2). Vigorous activity was the main contributor to the reduction in total physical activity levels, with a smaller proportion of adults in the ‘Type 2 diabetes + CVD’ group reaching the upper quartile of vigorous activity compared with the ‘No disease’ group (12.3% vs 23.7%) (table 2). Those with ‘Type 2 diabetes + CVD’ were 34%, 55% and 80% more likely to report low walking, moderate and vigorous activity levels, respectively, compared with the ‘No disease’ group (table 3). The proportion of adults who reported high TV viewing more than doubled in the ‘Type 2 diabetes + CVD’ group compared with the ‘No disease’ group (47.3% vs 20.3%) (figure 2). These results indicate that almost half the adults diagnosed with ‘Type 2 diabetes + CVD’ sit for >3 h/day watching TV. Almost three-quarters of the ‘No disease’ group report optimal sleep duration, but this proportion declined across cardiometabolic disease groups. The proportion of poor sleepers (<7 and >8 h) was higher in cardiometabolic disease groups compared with the ‘No disease’ group (figure 2). The proportion of adults who reported an ‘unhealthy phenotype’ increased across worsening cardiometabolic disease groups from 1.8% (No disease) to 10% (Type 2 diabetes + CVD), and the proportion reporting a ‘healthy phenotype’ decreased across groups (table 2).

**Table 3:** OR (95% CI) of reporting low walking, low moderate and low vigorous activity across cardiometabolic disease

	Low walking (min/day)	Low moderate activity (min/day)	Low vigorous activity (min/day)
No disease	1.00	1.00	1.00
CVD	1.08 (1.06 to 1.10)	1.18 (1.15 to 1.20)	1.38 (1.35 to 1.40)
Type 2 diabetes without CVD	1.16 (1.09 to 1.24)	1.35 (1.26 to 1.45)	1.53 (1.43 to 1.63)
Type 2 diabetes + CVD	1.34 (1.29 to 1.40)	1.55 (1.48 to 1.61)	1.88 (1.80 to 1.96)

All models adjusted for age, gender, body mass index, sociodemographic (Townsend Deprivation Index and ethnicity), smoking, alcohol and diet.

CVD, cardiovascular disease.

**Figure 3:**
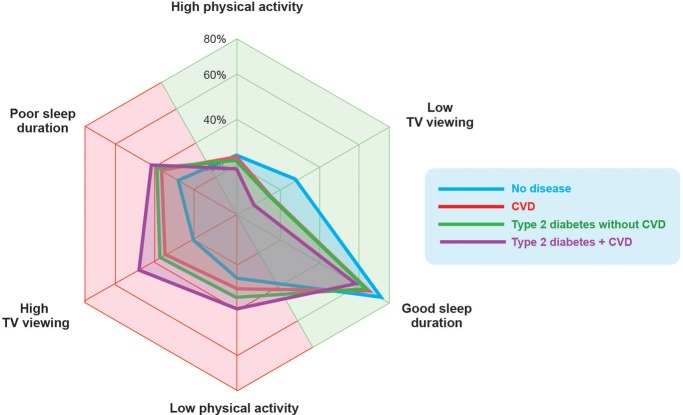
Radar chart showing the proportion of adults in each group who were categorised as either ‘high’ or ‘low’ for total physical activity or TV viewing, or ‘good’ or ‘poor’ for sleep duration. Green side indicates healthy non-diet lifestyle behaviours, whereas red side indicates unhealthy non-diet behaviours. There is a shift leftwards towards unhealthy behaviours with cardiometabolic disease.

Those with the most serious cardiometabolic disease profile (Type 2 diabetes + CVD) were 70% (OR (95% CI) 1.71 (1.64 to 1.78)), 90% (1.92 (1.85 to 1.99)) and 50% (OR 1.52, 95% CI 1.46 to 1.58) more likely to report low physical activity, high TV viewing and poor sleep duration, respectively, compared with the ‘No disease’ group (table 4). The odds of reporting all three unhealthy behaviours together was higher than reporting one of these lifestyle behaviours individually. Indeed, those in the ‘Type 2 diabetes + CVD’ group were three times more likely to report an ‘unhealthy phenotype’, (ie, low physical activity, high TV viewing and poor sleep duration) (OR=3.29 (95% CI 3.02 to 3.58)) even when controlling for age, gender, BMI, Townsend Deprivation Index, ethnicity, alcohol, smoking and meeting fruit/vegetable guidelines. The shift in unhealthy behaviours is visualised in figure 3 which shows the movement from healthy behaviours (green/right) to unhealthy behaviours (red/left).

**Table 4:** OR (95% CI) of reporting low physical activity, high sitting time, poor sleep and all behaviours combined across cardiometabolic disease

	Low physical activity	High TV viewing	Poor sleep	Low physical activity + high sitting+poor sleep
No disease	1.00	1.00	1.00	1.00
CVD	1.23 (1.20 to 1.25)	1.42 (1.39 to1.45)	1.37 (1.34 to1.39)	2.15 (2.03 to 2.28)
Type 2 diabetes without CVD	1.43 (1.34 to 1.53)	1.59 (1.49 to1.69)	1.38 (1.30 to1.47)	2.14 (1.85 to 2.48)
Type 2 diabetes + CVD	1.71 (1.64 to 1.78)	1.92 (1.85 to 1.99)	1.52 (1.46 to1.58)	3.29 (3.02 to 3.58)

All models adjusted for age, gender, body mass index, sociodemographic (Townsend Deprivation Index and ethnicity), smoking, alcohol and diet.

CVD, cardiovascular disease.

## Discussion

This is the largest cohort study to simultaneously assess diet, physical activity, TV viewing and sleep duration across cardiometabolic disease and non-disease groups. The results indicate that compared with disease-free individuals, (1) those with cardiometabolic disease report less physical activity, higher TV viewing and poorer sleep patterns; (2) non-diet unhealthy lifestyle behaviours were clustered, and people with ‘Type 2 diabetes + CVD’ were more than three times more likely to report low physical activity, high TV viewing and poor sleep duration at the same time and (3) people with cardiometabolic disease had changed their diet and were less likely to consume sugary foods. These results suggest that recommendations to change diet are reaching those with cardiometabolic disease, yet low physical activity, high TV viewing and poor sleep duration are significant, unaddressed, cardiometabolic risk factors.

### Strengths and weaknesses of the study

The present report holds strength in the large sample size and detailed measurements. The population-based design allows simultaneous presentation of behaviours in people with different stages of cardiometabolic disease, controlling for key factors including age, sex, socioeconomic status and BMI. However, the study is not without limitation. The response rate of the UK Biobank was low (5.5%), and a number of participants were excluded due to missing values, which may affect the generalisibility of the results to the UK population. The cross-sectional nature means we cannot infer whether these unhealthy lifestyle behaviours precede or were preceded by cardiometabolic disease. Over time, as the UK Biobank cohort progresses, longitudinal observations will be possible. Lifestyle behaviours were self-reported and not objectively measured. However, all questionnaires used validated self-reporting which allows these measures to be applied to large numbers of people. TV viewing is a poor surrogate for total daily sitting time, which is a more complete measure of sedentary behaviour, but could not be calculated from the UK Biobank questionnaire used. Residual confounding has to be acknowledged, as TV viewing is also influenced by other unhealthy behaviours such as increased snacking.^23^ That being said, TV viewing is a well established negative health behaviour, and these results have translational importance. With these limitations noted, the strengths of the present data mean that it has both scientific and practical implications.

### Relevance of findings to the field

*Diet*: People with type 2 diabetes were more likely to report changing their diet in the past 5 years and less likely to eat sugary foods compared with people with CVD or disease-free individuals. Dietary change is aligned to the current treatment advice for people with type 2 diabetes,^24^ and suggests that patients are acting on, or at least aware of, dietary advice. The food frequency questionnaire did not allow us to measure energy intake; therefore, it is possible that, although participants had changed their diet, they ate more. To address excess calorie intake, we have controlled for BMI as a means to manage excess calorie intake. By contrast with those with type 2 diabetes, people with CVD consume more sugary foods than people with diabetes, and are less likely to have changed their diet, as advised by current advice.^16^ Cardioprotective diets are promoted to reduce cholesterol and blood pressure in CVD.^25^ ^26^ Dietary advice remains the pillar of national guidelines for the management of type 2 diabetes, with evidence reporting that dietary changes can significantly improve glycemic control,^27^ and even reverse type 2 diabetes.^28^ ^29^ Analysis of the self-report diet behaviour from the UK Biobank cohort suggests that national dietary messaging is reaching those with type 2 diabetes, but not those with CVD.

*Physical activity*: Those with cardiometabolic disease report less physical activity than healthy counterparts, with vigorous activity being the largest contributor to this difference. A recent prospective meta-analysis confirmed a dose–response relationship between physical activity and type 2 diabetes risk, with the strongest associations seen with vigorous activity,^30^ which along with our results suggests that a lack of vigorous activity plays a major role in cardiometabolic disease. Acute vigorous activity stimulates greater peripheral glucose uptake compared with non-vigorous activity,^31^ which may explain the stronger association with vigorous activity and cardiometabolic disease. National physical activity guidelines encourage individuals to perform at least 150 min of moderate activity or 75 min of vigorous activity weekly.^13^ Based on these recommendations, 16% of the ‘No disease’ group do not perform adequate physical activity levels, and the percentage rises with cardiometabolic disease.

*TV viewing*: TV viewing was significantly higher in those with cardiometabolic disease compared with healthy adults. In a previous study, more than 3 h of TV viewing was strongly linked to all-cause mortality (RR 1.30; 95% CI 1.06 to 1.56)^6^ suggesting that those in the highest quartile of TV viewing in the UK Biobank cohort are exposing themselves to detrimental health consequences. A strong evidence base is accumulating for the negative health consequences of TV viewing. It is likely that low muscle activity has a direct physiological health impact as skeletal muscle is the largest insulin-sensitive organ in the body; however, TV viewing is also associated with other unhealthy behaviours such as snacking.^23^ As a result, it is consistently related to more adverse disease outcomes compared with daily sitting time and, therefore, should not be used as a surrogate marker for sedentary behaviour.

*Sleep duration*: These data reveal that across worsening cardiometabolic disease groups, the proportion of individuals reporting short or long sleep increases. We have defined optimal sleep duration as 7–8 h based on a recent review^10^ and our findings support previous observational studies which show a ‘U-shaped’ relationship between sleep duration and cardiometabolic disease. Sleep plays an integral role in metabolic regulation,^32^ with sleep restriction inducing insulin resistance and loss of circadian hormone changes. Indeed, the acute effects of sleep shortening are powerful. Sleep restricting healthy young men from 8 h/night to only 4 h/night for 1 week induces insulin resistance to a similar extent as people with type 2 diabetes.^33^ Sleep shortening also effects hormones that control appetite,^34^ elevating ghrelin and reducing leptin, which could explain the strong link between sleep deprivation, raised energy intake and weight gain.^34^ Persistent long sleep and increases in sleep duration over a 5-year period have been linked to higher type 2 diabetes incidence.^35^ However, the physiological impact of long sleep is yet to be fully understood. Although there will be a clear impact of long sleep on the opportunity to be physically active during wakefulness, more work is needed to explore the impact of normalising sleep in people who have long sleep. The benefits of improving sleep in people with existing cardiometabolic disease remains poorly described. However, given the potent effects of sleep on physiological function, these data highlight poor sleep as a potential therapeutic target in people with CVD and/or type 2 diabetes.

*Clustered lifestyle behaviours*: The results from this large population-based study also indicate an increased likelihood of reporting an ‘unhealthy phenotype’ encompassing low physical activity, high TV viewing and poor sleep duration, across worsening cardiometabolic disease groups. The ‘Type 2 diabetes + CVD’ group, who have a particularly poor prognosis, were more likely to report low physical activity, high TV viewing and poor sleep duration compared with all other groups, suggesting that these non-diet lifestyle behaviours may be exposing individuals to greater cardiometabolic risk beyond their disease. In the context of cardiometabolic disease and obesity, it is becoming increasingly common to combine physical activity and sitting as a joint association.^36^ ^37^ We have added sleep into our analysis, as we propose that all three behaviours are interdependent in their influence on metabolic control. Indeed, the clustering of these behaviours produces higher odds with cardiometabolic disease compared with individual behaviours. During sleep, there is a reduction in glucose utilisation, with an overall rise in plasma glucose.^38^ By contrast, throughout waking hours, physical activity stimulates peripheral glucose uptake,^39^ and is important for maintenance of euglycemia. Physical activity may be viewed as an activator of metabolism, whereas sleep is vital for restoring and resetting homeostasis, largely through energy regulation and repair. A lack of activation or restoration results in insulin resistance, a prominent feature of cardiometabolic disease.^40^ Individually, low physical activity, high TV viewing and poor sleep duration have negative metabolic consequences. The present data suggest that people with CVD and/or type 2 diabetes are more likely to be exposed to a potent negative ‘behavioural phenotype’ consisting of low physical activity, high TV viewing and poor sleep duration simultaneously.

### Implications for care teams, policymakers and people with cardiometabolic disease

Data from the UK Biobank suggest that poor non-diet lifestyle behaviours are prominent and unaddressed behaviours in the prevention and management of cardiometabolic disease. Our findings should not be taken to understate the importance of diet in cardiometabolic health. A balanced diet and weight management are critical, and efforts should continue to support people accordingly. However, the government recently described physical activity as a ‘key health priority in its own right’^41^ highlighting the importance of strategic planning with various sectors spanning transport, infrastructure and training of healthcare professionals. In 2014, Public Health England produced a framework to embed physical activity into the fabric of daily life,^42^ and the first national NHS prevention programme designed to prevent type 2 diabetes through diet and physical activity interventions.^43^ The present data reinforces the pressing need for evidence-based and effective programmes for physical activity for people with CVD and type 2 diabetes.

Awareness of the importance of sedentary behaviours in chronic disease lags physical activity, but is growing rapidly. In 2010, the department of health and the sedentary behaviour and expert working group recommended that more emphasis needed to be placed on minimising time spent sedentary.^44^ Indeed, NICE guidelines for type 2 diabetes prevention note the importance of reducing sitting time.^15^ Cross-sectional and longitudinal data provide evidence for the importance of TV viewing in cardiometabolic health,^6^ ^45^ ^46^ yet the importance of daily sitting time, which is a better marker of sedentary behaviour, is in question.^47^ Before guidelines and policies can be targeted towards sedentary behaviour, well-controlled intervention studies are urgently required to define the role of daily sitting in cardiometabolic disease. By contrast with physical activity and sitting, NICE guidelines for CVD and type 2 diabetes do not comment on sleep, despite the present data revealing that one in three of people with CVD, and nearly half the people with type 2 diabetes, sleep either too much or too little. Despite strong cross-sectional data, evidence is needed to define the impact of helping people with cardiometabolic disease achieve good sleep, to help inform guidelines and policy in this area.

A major finding from the present data was the clustering of physical activity, TV viewing and sleep behaviour. This is important as, to date, intervention studies have focused on changing a single lifestyle behaviour, with very few targeting multiple lifestyle behaviours.^48^ Given the clustering of these non-diet lifestyle behaviours, exploration of interventions incorporating physical activity, TV viewing and sleep, together may add value and should be the focus of future policies and programmes.

In summary, the preset data demonstrates that those with more advanced cardiometabolic disease undertake too little physical activity, have high TV viewing times and poor sleep duration, yet report important positive dietary changes within the past 5 years. These non-diet lifestyle behaviours are clustered, and indeed those with the worst cardiometabolic disease are three times more likely to display an ‘unhealthy behavioural phenotype’ compared with disease-free individuals, independent of age, gender, BMI and socioeconomic status. These novel data highlight that there remains a significant unaddressed behavioural phenotype of CVD and type 2 diabetes that places these people at excess risk of worsening cardiometabolic health. Strategies are urgently required to address physical activity, TV viewing and sleep to assist patients, care teams and policymakers in making effective decisions for the management and prevention of CVD and type 2 diabetes.
