# Supplementary material for: Cross-sectional study of diet, physical activity, television viewing and sleep duration in 233 110 adults from the UK Biobank; the behavioural phenotype of cardiovascular disease and type 2 diabetes
Source: BMJ Open. 2016 Mar 15;6(3):e010038. doi: 10.1136/bmjopen-2015-010038 (PMC4800116; doi:10.1136/bmjopen-2015-010038)
Supplement: Supplementary data [file bmjopen-2015-010038supp.pdf]

Online Supplement 1   Diseases included in the ‘CVD’ group

hypertension
heart/cardiac problem
peripheral vascular disease
venous thromboembolic disease
essential hypertension
gestational hypertension/pre-eclampsia
angina
heart attack/myocardial infarction
heart failure/pulmonary odema
heart arrhythmia
heart valve problem/heart murmur
cardiomyopathy
pericardial problem
stroke
transient ischaemic attack (tia)
subdural haemorrhage/haematoma
subarachnoid haemorrhage
leg claudication/ intermittent claudication
arterial embolism
pulmonary embolism +/- dvt
deep venous thrombosis (dvt)
peripheral neuropathy
ischaemic stroke
mitral valve disease
mitral regurgitation / incompetence
aortic valve disease
aortic regurgitation / incompetence
hypertrophic cardiomyopathy (hcm / hocm)
pericarditis
pericardial effusion
aortic aneurysm rupture
aortic dissection
aortic stenosis
brain haemorrhage

Online supplement 2 **General characteristics and lifestyle behaviours of the 'Other diseases' group (n=203,700) who were excluded from analysis.** Data shown in red for n=167,810 individuals who had information on physical activity, sitting and sleep behaviours.

	% within each disease group				
	**No Disease** *(n=103,993)*	**CVD** *(n=113,469)*	**Type 2 diabetes without CVD** *(n=4074)*	**Type 2 diabetes + CVD** *(n=11,574)*	**'Other diseases'** *(n=167,810)*
**SOCIO-DEMOGRAPHICS**					
**% Male**	47.0	53.3	63.6	68.0	**42.2**
**Age *(n)***	*103,993*	*113,469*	*4074*	*11,574*	***167,810***
60-73	28.6	57.4	53.9	66.4	**38.5**
**BMI *(n)***	*103,443*	*112,852*	*4048*	*11,478*	***167163***
≥30 (obese)	15.0	33.6	44.6	60.0	**18.8**
**Townsend deprivation quintile *(%)***	*103,861*	*113,323*	*4070*	*11,557*	***167,598***
5 (most deprived)	17.1	20.5	25.1	28.5	**18.2**
**DIET**					
**Dietary change in past 5 yrs**	*103,902*	*113,300*	*4070*	*11,555*	**167,620**
YES	28.9	45.9	75.5	75.3	**37.5**
**PHYSICAL ACTIVITY**					
**Total Physical activity a (MET.mins/wk)**	*103,993*	*113,469*	*4074*	*11,574*	***167,810***
≤918 (Low physical activity)	25.0	30.5	35.4	40.1	**28.2**
>918-1902	25.0	24.2	22.5	22.2	**24.4**
>1902-3706	25.0	22.2	20.7	19.7	**23.7**
>3706-19278 (High physical activity)	25.0	23.2	21.3	18.0	**23.8**
**TV VIEWING**					
**TV viewing a (hours/day)**	*103,993*	*113,469*	*4074*	*11,574*	***167,810***
≤1 (Low TV viewing)	26.6	16.2	15.4	10.5	**23.1**
>1-2	30.5	24.5	22.3	19.3	**28.2**
>2-3	22.6	24.5	24.1	22.8	**23.1**
>3 (High TV viewing)	20.3	34.8	38.1	47.3	**25.6**
**SLEEP**					
**Sleep duration c (hours/night)**	*103,993*	*113,469*	*4074*	*11,574*	***167,810***
<7 (Poor sleep duration)	21.3	26.4	27.0	27.5	**24.0**
7-8 (Good sleep duration)	73.4	64.6	62.1	58.6	**68.6**
>8 (Poor sleep duration)	5.3	9.1	10.8	13.9	**7.3**
**BEHAVIOURAL PHENOTYPE**					
UNHEALTHY	1.8	5.2	5.8	10.0	**3.1**
HEALTHY	4.5	2.4	1.9	1.2	**3.5**

Online supplement 3 **Prevalance data and logistic regression analysis for Total frequency and Total duration of physical activity from the UK Biobank questionnaire.** *Total frequency was calculated as the days for walking+moderate+activity and total duration was calculated as the minutes for walking+moderate+vigorous activity.*

TOTAL FREQ UENCY

A-proportion attaining physical activity quartiles in each disease group

	**HEALTHY**	**CVD**	**Type 2 diabetes without CVD**	**Type 2 diabetes +CVD**
**Total frequency physical activity (days)**	*103,993*	*113,469*	*4074*	*11,574*
≤7 (Low physical activity)	25.4	30.6	33.9	38.9
>7-11	28.7	28.0	27.5	25.7
>11-14	21.5	21.0	20.1	19/6
>14 (High physical activity)	24.4	20.3	18.5	15.8

B-Odds of reporting unhealthy behaviours

	**Low physical activity**	**High sitting time [TV viewing]**	**Poor sleep**	**Low Physical Activity + High Sitting + Poor Sleep**
**No Disease**	1.00	1.00	1.00	1.00
**CVD**	1.23 (1.21 to 1.26)	1.42 (1.39 to1.45)	1.37 (1.34 to1.39)	2.11 (1.99-2.23)
**Type 2 diabetes *without* CVD**	1.34 (1.25 to 1.44)	1.59 (1.49 to1.69)	1.38 (1.30 to1.47)	2.11 (1.83-2.44)
**Type 2 diabetes + CVD**	1.63 (1.56 to 1.71)	1.92 (1.85 to 1.99)	1.52 (1.46 to 1.58)	3.07 (2.82-3.34)

TOTAL DURATION

A-proportion attaining physical activity quartiles in each disease group

	**HEALTHY**	**CVD**	**Type 2 diabetes without CVD**	**Type 2 diabetes +CVD**
**Total duration physical activity (mins)**	*103,993*	*113,469*	*4074*	*11,574*
≤60 (Low physical activity)	27.8	34.1	39.5	44.7
>60-105	23.5	21.1	20.7	19.3
>105-180	26.4	23.4	20.2	19.7
>180 (High physical activity)	22.3	21.4	19.6	16.4

B-Odds of reporting unhealthy behaviour

	**Low physical activity**	**High sitting time [TV viewing]**	**Poor sleep**	**Low Physical Activity + High Sitting + Poor Sleep**
**No Disease**	1.00	1.00	1.00	1.00
**CVD**	1.23 (1.21 to 1.26)	1.42 (1.39 to1.45)	1.37 (1.34 to1.39)	2.12 (2.00 to 2.24)
**Type 2 diabetes *without* CVD**	1.43 (1.34 to 1.53)	1.59 (1.49 to1.69)	1.38 (1.30 to1.47)	2.13 (1.85 to 2.44)
**Type 2 diabetes + CVD**	1.72 (1.65 to 1.80)	1.92 (1.85 to 1.99)	1.52 (1.46 to1.58)	3.29 (3.04 to 3.57)

Online Supplement 4 **Socio-demographics of those who have missing data on physical activity, sitting or sleep and therefore excluded from analysis (n=60,938).**

	% within each disease group			
	**No Disease** *(n=23,515)* *20.7% missing*	**CVD** *(n=32,928)* *18.4% missing*	**Type 2 diabetes without CVD** *(n=1104)* *22.5% missing*	**Type 2 diabetes with CVD** *(n=3391)* *21.3% missing*
**SOCIO-DEMOGRAPHICS**				
**% Male**	39.2	40.6	53.8	53.0
**Age *(n)***	*23,515*	*32,928*	*1104*	*3391*
37-49	31.1	10.4	13.1	5.5
50-59	36.5	28.9	29.5	26.9
60-73	32.4	60.7	57.3	67.6
**BMI *(n)***	*22,903*	*32,651*	*1090*	*3356*
<18.5-24.9 (under and acceptable weight)	40.5	20.4	12.6	6.9
25-29.9 (overweight)	42.1	40.7	37.2	29.8
≥30 (obese)	17.4	39.0	50.3	63.4
**Townsend deprivation quintile *(n)***	*23,494*	*32,892*	*1099*	*3387*
1 (least deprived)	19.4	16.6	15.7	12.1
2	18.9	18.0	15.2	14.1
3	20.0	19.0	18.4	17.6
4	19.9	19.8	18.7	22.0
5 (most deprived)	21.8	26.7	32.0	34.3
**Ethnicity *(n)***	*22,828*	*32,574*	*1092*	*3365*
White/British	92.5	93.8	83.2	86.9
Mixed	0.7	0.5	0.5	0.7
Asian	2.5	2.0	9.8	6.5
Black African	2.2	2.4	3.8	3.8
Chinese	0.6	0.2	0.3	0.2
Other	1.4	1.0	2.4	1.8

Online Supplement 5 Dietary data from the food frequency questionnaire across cardio-metabolic disease groups.

	% within each disease group			
	**No Disease** *(n=103,993)*	**CVD** *(n=113,469)*	**Type 2 diabetes without CVD** *(n=4074)*	**Type 2 diabetes + CVD** *(n=11,574)*
**Oily fish**	*103,648*	*113,059*	*4,057*	*11,530*
Never	10.6	9.8	12.7	11.1
Less than once a week	35.3	30.2	31.9	30.6
Once a week	38.1	38.8	35.3	37.1
2-4 times a week	15.3	20.1	18.3	20.0
5-6 times a week	0.7	0.8	1.2	0.9
Once of more daily	0.2	0.3	0.6	0.4
**Processed meat**	*103,891*	*113,330*	*4,061*	*11,554*
Never	10.2	7.9	8.6	6.4
Less than once a week	30.9	29.6	27.2	25.0
Once a week	29.0	29.7	28.1	29.6
2-4 times a week	26.2	28.7	30.4	33.9
5-6 times a week	3.0	3.3	4.3	4.1
Once of more daily	0.7	0.9	1.4	1.1
**Poultry intake**	*103,916*	*113,349*	*4,069*	*11,551*
Never	5.8	4.0	5.1	3.7
Less than once a week	10.4	10.5	10.7	11.2
Once a week	36.0	36.3	34.2	35.3
2-4 times a week	45.6	46.9	46.8	47.1
5-6 times a week	1.9	2.1	2.7	2.3
Once of more daily	0.3	0.3	0.5	0.4
**Cheese intake**	*102,188*	*110,315*	*3,900*	*11,141*
Never	2.1	3.1	3.7	4.1
Less than once a week	14.9	18.5	23.2	21.2
Once a week	20.4	22.4	23.1	24.7
2-4 times a week	47.3	44.3	39.8	40.9
5-6 times a week	11.2	8.5	7.6	6.3
Once of more daily	4.0	3.1	2.6	2.8
**Salt intake**	*103,979*	*113,457*	*4,073*	*11,573*
Never/Rarely	56.5	58.4	54.3	55.5
Sometimes	28.0	26.3	28.7	28.0
Usually	11.3	10.8	12.0	11.9
Always	4.2	4.5	5.1	4.6
**Non-oily fish intake**	*103,705*	*113,135*	*4,055*	*11,516*
Never	4.7	4.0	5.7	4.8
Less than once a week	29.9	27.3	28.9	26.9
Once a week	49.8	51.1	47.9	49.9
2-4 times a week	15.0	16.8	16.4	17.6
5-6 times a week	0.5	0.5	0.8	0.5
Once of more daily	0.1	0.2	0.2	0.3
**Beef intake**	*103,790*	*113,154*	*4,057*	*11,534*

Never	11.6	9.3	12.1	9.3
Less than once a week	46.0	45.4	42.7	41.6
Once a week	31.8	33.0	32.6	34.9
2-4 times a week	10.5	12.0	12.2	13.7
5-6 times a week	0.2	0.2	0.4	0.3
Once of more daily	0.1	0.1	0.1	0.1
**Lamb/mutton intake**	*103,671*	*112,946*	*4,053*	*11,514*
Never	18.1	16.1	15.5	14.9
Less than once a week	58.1	56.2	52.8	51.8
Once a week	21.1	24.4	26.2	28.4
2-4 times a week	2.6	3.1	5.3	4.7
5-6 times a week	0.1	0.1	0.1	0.1
Once of more daily	0.0	0.0	0.0	0.1
**Pork intake**	*103,671*	*112,999*	*4,057*	*11,516*
Never	17.7	15.5	18.4	15.5
Less than once a week	58.4	56.7	51.9	51.8
Once a week	20.8	24.0	24.6	27.5
2-4 times a week	3.0	3.7	4.9	5.0
5-6 times a week	0.1	0.1	0.1	0.2
Once of more daily	0.0	0.0	0.1	0.1
**Milk type used**	*103,940*	*113,415*	*4,070*	*11,571*
Full cream	7.9	5.9	5.6	4.9
Semi-skimmed	66.3	64.0	65.6	66.2
Skimmed	18.1	21.9	21.4	22.2
Soya	3.7	3.5	3.5	2.5
Other type of milk	1.0	1.2	0.8	1.1
Never/rarely have milk	3.1	3.5	3.2	3.1
**Spread type**	*103,846*	*113,315*	*4,059*	*11,550*
Never/rarely use	11.3	10.7	10.4	8.4
Butter/spreadable	39.5	33.0	28.3	29.9
Flora pro active/benecol	0.4	0.6	0.6	0.7
Other type spread/marg	48.8	55.7	60.7	61.1
**Bread type**	*100,505*	*109,974*	*3,973*	*11,352*
White	24.2	27.9	23.8	29.7
Brown	12.6	12.9	14.0	13.0
Wholemeal/wholegrain	59.7	55.3	58.1	53.7
Other	3.5	3.9	4.2	3.6
**Cereal type**	*85,959*	*92,847*	*3,479*	*9,744*
Bran	17.1	16.7	16.1	16.9
Biscuit (e.g. Weetabix)	16.8	18.3	23.9	24.6
Oat (porridge)	23.9	26.8	29.2	29.0
Muesli	23.7	18.4	14.7	12.5
other	18.4	19.8	16.2	17.0
**Never eat**	*103,848*	*113,190*	*4,039*	*11,527*
Eggs or foods containing eggs	2.1	3.3	4.4	4.6
Dairy products	1.6	2.6	4.1	3.5
Wheat products	1.6	2.9	3.2	3.0
**Does your diet vary from week to week?**	*103,701*	*113,167*	*4,052*	*11,534*

Never/rarely	38.0	32.3	29.8	27.6
Sometimes	55.1	58.8	59.9	61.8
Often	6.9	8.9	10.3	10.6
**Bread intake (slices of bread each week) *mean* (SD)**	*12.1* (8.5)	*12.7* (8.7)	*14.4* (9.8)	*14.6* (9.3)
**Cereal intake (bowls of cereal per week) *mean* (SD)**	*4.5* (2.8)	*4.4* (2.8)	*4.8* (2.8)	*4.5* (2.8)
